# Engineering cell factories for producing building block chemicals for bio-polymer synthesis

**DOI:** 10.1186/s12934-016-0411-0

**Published:** 2016-01-21

**Authors:** Yota Tsuge, Hideo Kawaguchi, Kengo Sasaki, Akihiko Kondo

**Affiliations:** Organization of Advanced Science and Technology, Kobe University, 1-1 Rokkodai, Nada, Kobe, 657-8501 Japan; Department of Chemical Science and Engineering, Graduate School of Engineering, Kobe University, 1-1 Rokkodai, Nada, Kobe, 657-8501 Japan; Biomass Engineering Program, RIKEN, 1-7-22 Suehiro-cho, Tsurumi-ku, Yokohama, Kanagawa 230-0045 Japan

**Keywords:** Metabolic engineering, Bio-polymers, Lactic acid, Succinic acid, Adipic acid, Putrescine, Cadaverine, High-performance polymers, *Corynebacterium glutamicum*, *Escherichia coli*

## Abstract

Synthetic polymers are widely used in daily life. Due to increasing environmental concerns related to global warming and the depletion of oil reserves, the development of microbial-based fermentation processes for the production of polymer building block chemicals from renewable resources is desirable to replace current petroleum-based methods. To this end, strains that efficiently produce the target chemicals at high yields and productivity are needed. Recent advances in metabolic engineering have enabled the biosynthesis of polymer compounds at high yield and productivities by governing the carbon flux towards the target chemicals. Using these methods, microbial strains have been engineered to produce monomer chemicals for replacing traditional petroleum-derived aliphatic polymers. These developments also raise the possibility of microbial production of aromatic chemicals for synthesizing high-performance polymers with desirable properties, such as ultraviolet absorbance, high thermal resistance, and mechanical strength. In the present review, we summarize recent progress in metabolic engineering approaches to optimize microbial strains for producing building blocks to synthesize aliphatic and high-performance aromatic polymers.

**Electronic supplementary material:**

The online version of this article (doi:10.1186/s12934-016-0411-0) contains supplementary material, which is available to authorized users.

## Background

Since the discovery and commercialization of synthetic polymers, these materials have become essential for everyday life [1]. Currently, almost all polymer building block chemicals are produced by petroleum-based chemical processes. Although such processes are able to produce a wide variety of materials at relatively low cost, these methods are inherently non-sustainable and have detrimental impacts on the environment. For these reasons, there is increasing global demand to replace petroleum-based production processes with microbial synthetic procedures that utilize renewable resources. The bio-based production of polymer building block chemicals is also advantageous because the synthetic reactions can be performed at near-standard temperatures and pressures, which markedly reduces the amount of required energy.

Despite the clear advantages of bio-based chemicals, poly-l-lactic acid (PLLA) is to date perhaps the only good example of successful industrialization of a 100 % bio-based polymer [2]. The main limitation for shifting to microbial synthetic processes is the high production cost. Specifically, the volumetric and specific productivities, and yields of target compounds by microbial fermentation, are often much lower than those obtained by chemical synthetic processes. For these reasons, the engineering of microbial strains that rapidly reach high-cell densities and have productivities and yields of target compounds at close to the theoretical maxima is needed for the commercialization of bio-based products. Genomic sequencing has opened the door for systems metabolic engineering for many industrially important microorganisms, such as *Escherichia coli*, *Corynebacterium glutamicum*, and *Saccharomyces cerevisiae*. In combination with genetic engineering tools and knowledge of metabolism and pathway regulation, sequence information has facilitated the rational design of strains with high productivity and yields of target compounds [3–8]. In addition, recent development of -omics techniques and computational tools have drastically accelerated the process of strain optimization [9].

In this review, we summarize recent knowledge of gene targets for metabolic engineering that efficiently convert glucose to building block chemicals (such as d-lactic acid, succinic acid, adipic acid, putrescine, and cadaverine) performed primarily in *C. glutamicum* and *E. coli*, that permit the synthesis of aliphatic polymer. We then expand the scope of our discussion to the production of other building block chemicals (such as d-phenyllactic acid, 3-amino-4-hydroxybenzoic acid, and cinnamic acid) for the synthesis of aromatic polymers.

## Building block chemicals for aliphatic polymer synthesis

### d-Lactic acid

**Fig. 1:**
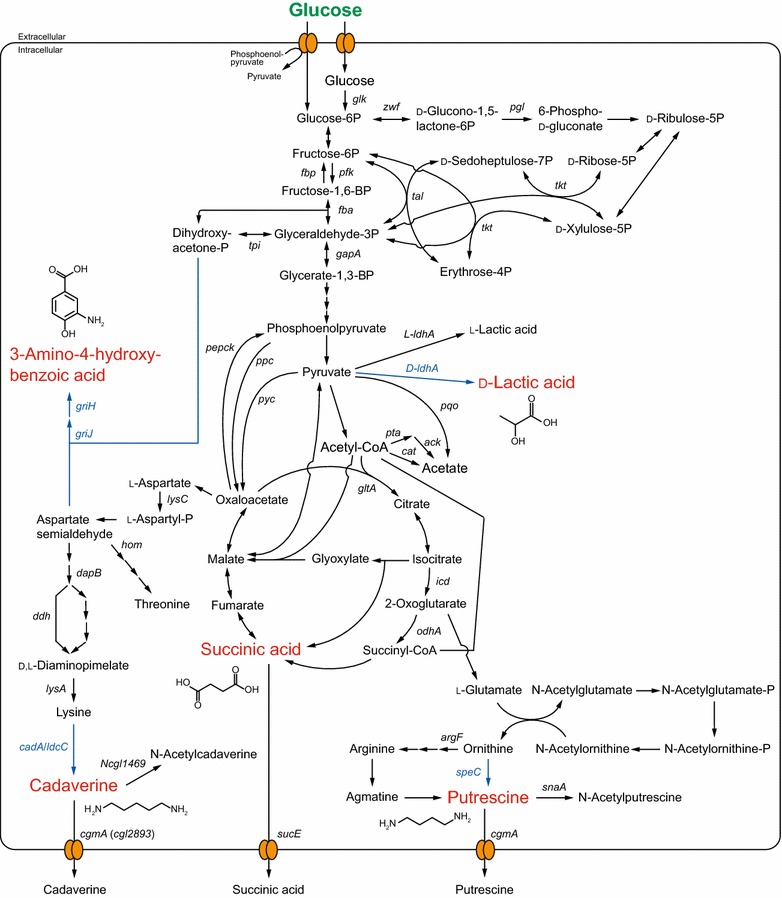
Schematic representation of the metabolic pathway in *C. glutamicum* for the production of building block chemicals (d-lactic acid, succinic acid, putrescine, cadaverine, and 3, 4-AHBA) for polymer synthesis. Substrate and target chemicals are presented in *green* and *red*, respectively. Heterologous *genes* and *lines* indicating the corresponding reactions are shown in *blue*. The deletion, overexpression, or nucleotide substitution of the *genes* indicated in the metabolic pathways leads to improved production of the target chemicals. Corresponding enzymes and functions are listed in Additional file 1: Table S1

Lactic acid (2-hydroxypropanoic acid) is synthesized in one step from pyruvate, the end product of the glycolytic pathway, by lactate dehydrogenase (LDH), which is encoded by the *ldhA* gene (Fig. 1). Lactic acid has two optical isomers, l- and d-lactic acid, whose synthesis is dependent on the chiral-specific L- or D-LDH enzyme expressed by the microorganism. The optical purity of lactic acid is critical for its polymeric characteristics, as small amounts of optical impurities drastically change properties such as crystallinity, which directly affects thermal resistance [10]. PLLA is the most common bio-based and biodegradable polymer, and is frequently utilized as a film due to its high transparency [11]. However, because this polymer has low melting and glass transition temperatures, PLLA’s use in practical applications is limited [12]. Stereocomplex PLA (scPLA) composed of both PLLA and poly-d-lactic acid can circumvent this defect [12]. To produce high-quality scPLA, microbial strains that produce l- and d-lactic acid with high optical purity are required. As the microbial production of l-lactic acid is well established [13, 14], this section focuses on recent advances in metabolic engineering approaches for producing optically pure d-lactic acid.

*Corynebacterium glutamicum*, which is well known as a producer of amino acids such as glutamate and lysine [15, 16], displays arrested cell growth under oxygen-deprived conditions and also produces the organic acids l-lactate, succinate, and acetate [17]. The culturing in mineral salts medium of *C. glutamicum* at high cell-density under oxygen-deprived conditions led to the high volumetric productivity of organic acids [18]. Introduction of the D-LDH-encoding *Lactobacillus delbrueckiildhA* gene into a *C. glutamicum* mutant lacking the endogenous L-LDH-encoding gene yielded a strain that produced 120 g/L d-lactic acid of greater than 99.9 % optical purity (Table 1) [19]. Further disruption in this strain of the endogenous *ppc* gene (encoding phosphoenolpyruvate carboxylase, the primary source of succinic acid production) decreased succinic acid yield, but also reduced the glucose consumption rate [32]. The simultaneous overexpression of five glycolytic genes, namely *glk* (encoding glucokinase), *gapA* (encoding glyceraldehyde phosphate dehydrogenase), *pfk* (encoding phosphofructokinase), *tpi* (encoding triosephosphate isomerase), and *fba* (encoding bisphosphate aldolase), compensated for this impairment of glucose consumption and enabled the engineered *C. glutamicum* strain to produce 195 g/L d-lactic acid, corresponding to a yield of 1.80 mol/mol glucose (Fig. 1; Table 1) [20].

**Table 1:** Summary of microbial production of polymer building block chemicals from glucose with notable productivities

Monomer	Organism	Titer (g/L)	Yield (mol_product_/mol_substrate_)	Cultivation time (h)	References
d-Lactic acid	*C. glutamicum* R Δ*ldhA*/pCRB204	120	1.73	30^a^	[19]
	*C. glutamicum* R LPglc279/pCRB215	195	1.80	80^a^	[20]
	*E. coli* B0013-070B	122	1.69	28	[21]
Succinic acid	*C. glutamicum* R Δ*ldhA*/pCRA717	146	1.40	46^a^	[22]
	*C. glutamicum* ATCC13032 BOL-3/pAN6-*gap*	134	1.67	54^a^	[23]
	*C. glutamicum* ATCC13032 SA5	109	1.32	98^a^	[24]
	*E. coli* AFP111/pTrc99A-*pyc*	99	1.10	76	[25]
	*E. coli* KJ134	72	1.53	95	[26]
	*E. aerogenes* ES08 Δ*ptsG*	55	1.32	60	[27]
Putrescine	*E. coli* XQ52/p15SpeC	24	–	32	[28]
	*C. glutamicum* ^b^	19	0.33	34	[29]
Cadaverine	*C. glutamicum* DAP-16	88	0.50	40	[30]
	*E. coli* XQ56/p15CadA	9.6	0.07	30	[31]

^a^Only production phase

^b^Strain name unknown

**Fig. 2:**
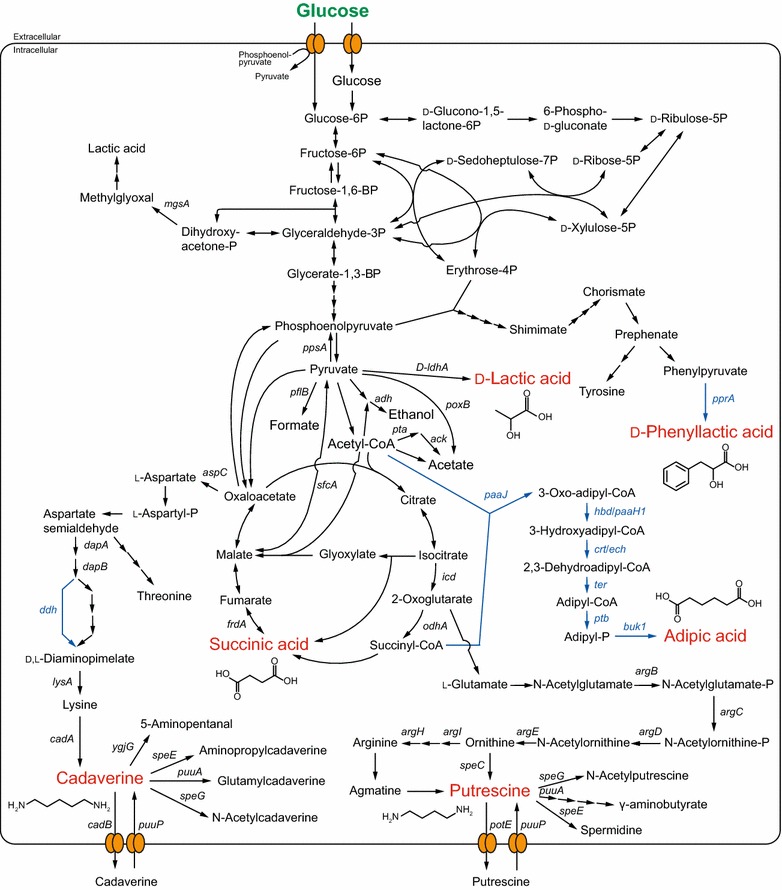
Schematic representation of the metabolic pathway in *E. coli* for the production of building block chemicals (d-lactic acid, succinic acid, adipic acid, putrescine, cadaverine, and phenyllactic acid) for polymer synthesis. Substrate and target chemicals are presented in *green* and *red*, respectively. Heterologous *genes* and *lines* indicating the corresponding reactions are shown in *blue*. The deletion, overexpression, or nucleotide substitution of the *genes* indicated in the metabolic pathways leads to improved production of the target chemicals. Corresponding enzymes and functions are listed in Additional file 1: Table S1

*Escherichia coli* naturally produces optically pure d-lactic acid and has many advantages as a host for microbial production, such as simple nutritional requirements and well-established systems for genetic manipulation [33]. However, *E. coli* performs mixed-acid fermentation, in which the principal products are d-lactate, succinate, acetate, formate, and ethanol [33]. For this reason, attempts to increase d-lactic acid production by *E. coli* have mainly focused on minimizing the production of by-products without decreasing the growth or sugar consumption rates [34–37]. For example, Zhou and colleagues metabolically engineered *E. coli* for d-lactic acid production by the deletion of *ackA* (encoding acetate kinase), *pta* (encoding phosphotransacetylase), and *poxB* (encoding pyruvate oxidase) to minimize acetate production, in addition to deleting *adhE* (encoding alcohol dehydrogenase) to prevent ethanol fermentation, *ppsA* (encoding phosphoenolpyruvate synthase) and *pflB* (encoding pyruvate formate lyase) to promote pyruvate accumulation, and *frdA* (encoding fumarate reductase) to prevent succinic acid accumulation (Fig. 2) [38]. The resultant strain produced highly optically pure (>99.9 %) d-lactic acid at concentrations reaching 125 g/L in 39 h, corresponding to a yield of 0.87 g/g glucose. This group subsequently demonstrated that replacing the *ldhA* gene promoter with the λ P_*R*_ and P_*L*_ promoters and shifting the temperature from 33 to 42 °C (thereby permitting the strict separation of the growth and d-lactic acid production phases) improved d-lactate productivity by minimizing the inhibitory effect of the produced d-lactate on cell growth and increasing the activity of LDH [21]. Using this approach, the engineered strain produced 122 g/L d-lactic acid in 28 h at a yield of 0.84 g/g glucose (Table 1).

### Succinic acid

Succinic acid (butanedioic acid) is a dicarboxylic acid of the tricarboxylic acid (TCA) cycle (Fig. 1) and is utilized as a building block for several commercially important polymers, such as polybutylene succinate adipate [39, 40]. Moreover, in combination with diamines, putrescine, and cadaverine, succinic acid can also be used to produce 100 % bio-based nylon materials [41, 42]. Therefore, the potential of bio-based processes to replace chemical-based succinic acid production has been extensively studied [6, 25, 42–44].

Several microorganisms, including *Anaerobiospirillum succiniciproducens* and *Actinobacillus succinogenes*, naturally produce high amounts of succinic acid as an end-product of anaerobic fermentation [45–48]. Under anaerobic conditions, succinic acid is produced mainly from phosphoenolpyruvate and pyruvate through anapleotic pathways and the reductive branch of the TCA cycle via the intermediates oxaloacetate, malate, and fumarate (Fig. 1) [19, 49]. *Corynebacterium glutamicum* produces small amounts of succinic acid under anaerobic conditions. However, by deleting the *L*-*ldhA* gene and overexpressing the *pyc* gene (encoding pyruvate carboxylase), Okino et al. engineered *C. glutamicum* to produce 146 g/L succinic acid with a yield of 1.40 mol/mol glucose in a two-stage (aerobic growth and anaerobic fed-batch production) system [22]. Despite this marked increase in succinic acid production, a large amount of acetate was still produced as a by-product. Additional deletions of genes in acetate-producing pathways, including *pta* (encoding phosphotransacetylase), *ackA* (encoding acetate kinase), *cat* (encoding acetyl-CoA:CoA transferase), and *pqo* (encoding pyruvate oxidoreductase) [50], in combination with the overexpression of a mutant *pyc*^*P458S*^ gene, *fdh* gene (encoding formate dehydrogenase) from *Mycobacterium vaccae*, and *gapA* gene, further increased the yield of succinic acid to 1.67 mol/mol glucose, corresponding to a titer of 133.8 g/L (Table 1) [23]. Overexpression of *gltA* (encoding citrate synthase) helped to channel more carbon towards the glyoxylate pathway, and overexpression of the previously identified *sucE* gene (encoding succinate exporter) in *C. glutamicum* [51, 52] was also employed. In combination, overexpression of these two genes provided 9 and 19 % increases in succinate yield and productivity, respectively [24].

*Escherichia coli* uses mixed-acid fermentation under anaerobic conditions to generate various products, including succinate, d-lactate, acetate, formate, and ethanol, as described above. Most studies aimed at increasing succinic acid production by *E. coli* have focused on eliminating the production of by-products and balancing the cellular redox state [43]. Using this approach, an *E. coli* strain was engineered for producing succinic acid in a single-step fermentation strategy. Specifically, deletion of the *ldhA*, *adhE* (encoding alcohol dehydrogenase), *pflB*, *focA* (encoding formate transporter), *pta*-*ackA*, *mgsA* (encoding methylglyoxal synthase), *poxB* (encoding pyruvate oxidase), and combined deletion of *aspC* (encoding aspartate aminotransferase) and *sfcA* (encoding malic enzyme) genes markedly reduced by-product formation and stimulated the reductive pathway, resulting in the production of 71.5 g/L succinic acid with a yield of 1.53 mol/mol glucose (Fig. 2; Table 1) [26].

Although the above-described *C. glutamicum* and *E. coli* strains were metabolically engineered to efficiently produce succinic acid, these strains are limited to growth at neutral pH conditions due to their sensitivity to acid stress [7, 53]. The production of organic acids is ideally performed at low pH to avoid the need for alkali solutions for pH neutralization during fermentation, and more importantly, to reduce the costs of downstream purification, which typically requires large quantities of acid [54, 55]. *Saccharomyces cerevisiae* is a promising candidate to overcome this limitation because of its high tolerance to acid stress, as demonstrated by its ability to grow at pH 3.0 [56, 57]. However, even after extensive metabolic engineering, the maximum succinic acid titer generated by *S. cerevisiae* remained too low for viable commercial production [58, 59]. Recently, Tajima and colleagues showed that the metabolic engineering of a newly isolated Gram-negative bacterium, *Enterobacter aerogenes* AJ110637, led to a producer of succinic acid under low pH conditions. This bacterium rapidly assimilated glucose at pH 5.0 [60]. Because the strain produced succinate, lactate, formate, and acetate (in addition to ethanol and 2, 3-butanediol) by mixed-acid fermentation, four genes [*ldhA*, *adhE*, *pta*, and *budA* (encoding α-acetolactate decarboxylase)] involved in by-product formation were deleted to minimize by-product accumulation. The gene-deleted strain was further engineered by overexpression of the *pck* gene (encoding phosphoenolpyruvate carboxykinase) from *A. succinogenes* and the *pyc* gene (encoding pyruvate carboxylase) from *C. glutamicum*, providing production of 11.2 g/L succinic acid at pH 5.7 [61]. However, this titer was 50 % lower than that obtained at pH 7.0, demonstrating that lowering the culture pH negatively impacts succinic acid production. To increase the acid tolerance of this strain, this group attempted to maximize adenosine-5′-triphosphate (ATP) yield, as used in *E. coli* [62, 63]. To accomplish this, the *ptsG* gene (encoding glucose-phosphotransferase system permease) was deleted, together with individual overexpression of the *pck* gene from *A. succinogenes* instead of the two anapleotic pathway genes. Further deletion of *poxB* and *pflB*, along with overexpression of *frdABCD* (encoding fumarate reductase), resulted in the production of 55.4 g/L succinic acid at pH 5.7 (Table 1) [27].

### Adipic acid

Polyamide, commonly known as nylon, has recurring amide groups linking the monomers to chains, and shows high durability and strength. More than 6 million tons of nylon are produced annually, and this polymer is considered indispensable for modern life [64]. The most common commercial polyamides are nylon-6 and nylon-6, 6, which account for more than 90 % of the global market. Adipic acid (1, 4-butanedicarboxylic acid) is a building block dicarboxylic acid that permits (in combination with 1, 6-hexamethylenediamine) the synthesis of nylon-6, 6 polyamide [65]. Currently, nearly all adipic acid is commercially produced petro-chemically from benzene via cyclohexane [66], and approximately 65 % of adipic acid is used for synthesizing nylon-6, 6 polyamide [67]. Therefore, the development of bio-based methods for adipic acid production in place of petroleum-based processes is expected to allow the synthesis of “green” polymers. Although a cellular metabolic degradation pathway for adipic acid has been described in *Pseudomonas* and *Acinetobacter* sp. [68, 69], the biosynthetic route towards adipic acid from the carbon source, such as glucose, through central metabolic pathways has not been reported. Until recently, bio-based adipic acid was obtained through the chemical conversion of the precursors glucaric acid and *cis*, *cis*-muconic acid, which can be biologically synthesized in metabolically engineered *E. coli* via myo-inositol or through the shikimate pathway from glucose [67]. Yu and colleagues described direct production of adipic acid from glucose by reversal of the adipate degradation pathway [70]. Specifically, adipic acid was produced in six enzymatic steps from acetyl-CoA and succinyl-CoA through 3-oxoadipyl-CoA, 3-hydroxyadipyl-CoA, 2, 3-dehydroadipyl-CoA, adipyl-CoA, and adipyl-phosphate (Fig. 1). To construct the complete pathway from acetyl-CoA and succinyl-CoA to adipic acid in *E. coli*, this group selected six enzyme genes for overexpression from *E. coli*, *Clostridium acetobutylicum*, and *Euglena gracilis*, and performed multiple gene deletions to minimize the accumulation of by-products and direct carbon flux towards the two precursors, acetyl-CoA and succinyl-CoA (Fig. 2). When engineered using this approach, the recombinant *E. coli* strain produced 639 µg/L adipic acid [70]. Deng and Mao later reported that the moderately thermophilic soil bacterium *Thermobifida fusca* naturally possesses the genes responsible for converting acetyl-CoA and succinyl-CoA to adipic acid; this bacterium produces 2.23 g/L adipic acid after 72 h cultivation at 55 °C [71].

### Putrescine

Diamine is a building block chemical for synthesizing polyamide with dicarboxylic acid. To achieve the production of 100 % bio-based polyamide, an efficient microbial production of diamines that replaces traditional petroleum-based synthesis is required. 1, 6-Hexamethylenediamine, a building block for synthesizing nylon-6, 6, has not been produced by microbial fermentation. However, diamines with different carbon atom numbers also can be used for synthesizing bio-based polyamide. For example, a four-carbon diamine, putrescine (1, 4-diaminobutane), is a promising target for microbial fermentation; this compound is industrially produced by chemical synthesis via the addition of hydrogen cyanide to acrylonitrile through succinonitrile [72]. Nylon-4, 6 (distributed by DSM as Stanyl^®^, which is synthesized from putrescine and adipic acid) has been demonstrated to possess mechanical and physical properties comparable, or even superior, to those of nylon-6, 6 in terms of melting point, glass transition temperature, tensile strength, solvent resistance, and rate of crystallization [73]. In addition, polymerization with sebacic acid, a ten-carbon dicarboxylic acid derived from castor plant oil, yields a 100 % bio-based nylon-4, 10; this polymer, which is distributed as by DSM as EcoPaXX^®^, has a high melting point and high crystallization rate and has been used as an engineering plastic [74].

Putrescine can be synthesized from two alkaline amino acids, l-ornithine or its downstream product l-arginine, through a single decarboxylation reaction catalyzed by ornithine decarboxylase or arginine decarboxylase, respectively (Figs. 1, 2) [29]. To date, the highest titer of microbial-produced putrescine was achieved using an engineered strain of *E. coli*. In this strain, designated XQ52/p15SpeC, *potE* (encoding putrescine/ornithine antiporter) was overexpressed in combination with the deletion of *puuP* (encoding putrescine importer) and of genes encoding enzymes of competitive and degradation routes for putrescine (including *puuA* (encoding glutamate-putrescine ligase), *speE* (encoding spermidine synthase), *speG* (encoding spermidine acetyltransferase), and *argI* (encoding a component of ornithine transcarbamylase) (Fig. 2). In addition, the native promoters of key biosynthetic genes [*argECBH* operon, *argD* (encoding N-acetyl-ornithine aminotransferase) and *speC* (encoding ornithine decarboxylase)] were replaced with stronger promoters, and *argR* (encoding a transcriptional repressor) and *rpoS* (encoding a stress-responsive RNA polymerase sigma factor) also were deleted (Fig. 1). The resultant strain was capable of producing 24.2 g/L putrescine (Table 1) [28].

*Corynebacterium glutamicum* also is a promising host for putrescine production because of this species’ capability of large-scale production of l-glutamic acid [75], as well as higher tolerance to putrescine compared to *E. coli* and *S. cerevisiae* [76]. Although the putrescine metabolic pathway has not been identified in *C. glutamicum*, introduction of the *speC* gene from *E. coli* enabled *C. glutamicum* to synthesize putrescine [76]. Recently, the Wendisch group has energetically identified engineering targets for increasing putrescine production in *C. glutamicum* (Fig. 1). This group demonstrated that deletion of *argF* (encoding ornithine transcarbamylase) and *argR* was effective for increasing putrescine production due to an increase in the ornithine supply; however, *argF* deletion resulted in arginine auxotrophy. This problem was circumvented by the fine-tuning of *argF* expression through modifications of the promoter, translational start codon, and ribosome-binding site, resulting in a 60 % increase in putrescine production [77]. Furthermore, this group also identified a gene responsible for putrescine acetylation, *snaA*, and showed deletion of *snaA* minimized the generation of acetylputrescine as a by-product, resulting in a further 41 % increase in putrescine production [78]. The Wendisch group also identified a putative putrescine transporter, CgmA, which was first identified as a cadaverine transporter (Cg2893; see cadaverine section), and demonstrated that overexpression of the *cgmA* gene increased putrescine production by 24 %, although *cgmA* overexpression in a *snaA*-deletion strain did not result in further increases in putrescine production [77]. The decreased activity of 2-oxoglutarate dehydrogenase (ODH) in *C. glutamicum* is associated with glutamate overproduction [79, 80]. To examine the effect of excess glutamate on putrescine production by *C. glutamicum*, ODH activity was decreased five-fold. This effect required replacement of the start codon of a gene (*odhA*) that encodes a subunit of the ODH complex, as well as mutating the gene (*odhI*) encoding an inhibitory protein for ODH complex (creating a Thr15-to-Ala substitution in OdhI to remove a phosphorylation site, because phosphorylated OdhI inhibits the function of ODH) [81, 82]. This genetic engineering strategy improved putrescine production by 28 %, corresponding to a yield of 0.26 g/g glucose, a value that is higher than that achievable with *E. coli* [83].

Very recently, park and colleagues reported the metabolic engineering of a strain of *C. glutamicum* capable of producing 92.5 g/L l-arginine in fed-batch fermentation [84]. Construction of this strain involved removing regulatory repressors of the *arg* operon, optimizing nicotinamide adenosine dinucleotide phosphate levels, disrupting the l-glutamate exporter gene (*cgl1270*) to increase production of the l-arginine precursor, and flux optimizing the rate-limiting l-arginine biosynthetic reactions. This engineered strain would be suitable for overproducing ornithine; thus, the strain might be rendered useful for the efficient production of putrescine by introducing the decarboxylase-encoding gene and metabolic engineering of targets as described above.

### Cadaverine

Cadaverine (1, 5-diaminopentane), a five-carbon diamine, is another candidate for the synthesis of “green” nylon [41]. Cadaverine is synthesized by the one-step decarboxylation of l-lysine, which is produced from oxaloacetate of the TCA cycle (Figs. 1, 2). The microbial production of cadaverine was first demonstrated in a metabolically engineered strain of *C. glutamicum*. Although *C. glutamicum* lacks the decarboxylase gene for converting l-lysine to cadaverine, the introduction of *cadA* (encoding lysine decarboxylase) from *E. coli*, in combination with the deletion of the endogenous *hom* gene (which encodes a homoserine dehydrogenase), enabled the production of 2.6 g/L cadaverine [85]. *E. coli* also has been engineered to produce 9.6 g/L cadaverine by deleting genes of the cadaverine degradation pathway and overexpressing lysine pathway genes (Fig. 2) [31].

*Corynebacterium glutamicum* is so far a superior host for large-scale, bio-based cadaverine production because of its ability to produce large quantity of l-lysine [86]. Several genetic mutations (*lysC*^*T311I*^ encoding aspartokinase, *hom*^*V59A*^, and *pyc*^*P458S*^) have been identified that improve lysine production through the deregulation of feedback resistance [87]. Recently, the Wittmann group extensively examined cadaverine production by *C. glutamicum*. In addition to the mutations of *lysC*^*T311I*^, *hom*^*V59A*^, and *pyc*^*P458S*^, chromosomal overexpression of the lysine pathway genes *dapB* (encoding dihydrodipicolinate reductase) and *pyc* by replacing the promoters, integration of a second copy of *ddh* (encoding diaminopimelate dehydrogenase) and *lysA* (encoding diaminopimelate decarboxylase), and deletion of *pepck* (encoding phosphoenolpyruvate carboxykinase) markedly increased cadaverine production (Fig. 1) [88]. In that study, another lysine carboxylase-encoding gene from *E. coli*, *ldcC*, was employed instead of *cadA* because the LdcC protein prefers neutral pH [89]. However, approximately 20 % of the intracellular cadaverine produced by the resultant strain was acetylated [88]. The Wittmann group therefore identified a gene responsible for cadaverine acetylation (*Ncgl1469* encoding diaminopentane acetyltransferase) in *C. glutamicum* by targeted, single-gene deletion of 17 potential N-acetyltransferases [90]. Notably, the identified gene shared low homology with the *snaA* gene, responsible for putrescine acetylation. Deletion of the *Ncgl1469* gene increased the yield of cadaverine by 11 %. Genome-wide transcriptional analysis led to the further identification of an exporter gene (*cg2893*), which was later identified as a putrescine transporter (CgmA; see putrescine section). Cadaverine secretion was improved by 20 % when *cg2893* was overexpressed [91]. Further metabolic engineering of *C. glutamicum* was conducted to replace the common ATG start codon of the *icd* gene (encoding isocitrate dehydrogenase) with the rare GTG (generating a variant designated *icd*^*GTG*^) to increase the flux through the anapleotic pathway, and to overexpress the *tkt* operon genes *zwf* (encoding glucose-6-phosphate dehydrogenase), *tal* (encoding transaldolase), *tkt* (encoding transketolase), *opcA* (encoding a putative subunit of glucose-6-phosphate dehydrogenase), and *pgl* (encoding 6-phosphogluconolactonase) by promoter exchange (Fig. 1). The resultant strain produced 88 g/L cadaverine, corresponding to a molar yield of 50 % (Table 1) [30]. The cadaverine produced by this strain was polymerized with sebacic acid to synthesize 100 % bio-polyamide (nylon-5, 10), which displayed a comparable melting point (215 °C) and glass transition temperature (50 °C), and even higher transparency, to that of the petrochemical polymers nylon-6, and nylon-6, 6 [30].

## Building block chemicals for aromatic polymer synthesis

The above sections focused on building block chemicals for synthesizing aliphatic polymers. In this final section, we describe the production of aromatic chemicals that potentially can be used to synthesize high-performance plastics that possess desirable properties such as ultraviolet (UV) absorbance, higher thermal resistance, and mechanical strength compared to aliphatic polymers. These next-generation bio-polymers may be applicable for the production of performance fabrics and electronics, and for use in the automobile and air industries. To be used for applications in these fields, the materials must have a glass transition temperature close to 200 °C, in addition to high mechanical strength and Young’s modulus [92]. To address this issue, the production of aromatic “bio-monomers” by microbial fermentation or bioconversion has been the subject of considerable research in the past decade, although the productivity of most chemicals remains limited.

d-phenyllactic acid (d-PhLA), one candidate precursor, is synthesized through the shikimate pathway via erythrose-4-phosphate, itself a product of the pentose phosphate pathway (Fig. 2). Optically pure d-PhLA was produced from glucose at a titer of 29 g/L by a recombinant *E. coli* strain expressing the *pprA* gene (encoding phenylpyruvate reductase) from *Wickerhamia fluorescens* [93]. More recently, d-PhLA was produced from the lignocellulosic biomass of kraft pulp [94] and pretreated bagasse [95] in a single-pod reaction of simultaneous saccharification and fermentation.

Cinnamic acid is a phenylalanine derivative that also has been produced from glucose by recombinant *Pseudomonas putida* [96] and *Streptomyces lividans* [97] overexpressing the *pal* genes (encoding phenylalanine ammonia lyase) from *Rhodosporidium toruloides* and *Streptomyces maritimus*, respectively (Fig. 1). The hydroxycinnamate derivatives of 4-hydroxycinnamic acid (*p*-coumaric acid) [98] and 3, 4-dihydroxycinnamic acid (caffeic acid) [99] were used as building blocks for the synthesis of aromatic bio-based polyesters with a glass transition temperature of 169 °C. It has also been demonstrated that the chemocatalytic processing of bio-monomers confers multiple properties to the resulting biopolymers. For instance, a bio-based copolymer formed from caffeic acid and *p*-coumaric acid showed strong adhesive characteristics [99], and caffeic acid was recently produced from glucose by recombinant *E. coli* [100].

3-Amino-4-hydroxybenzoic acid (3, 4-AHBA) serves as a subunit of poly-benzoxazole [101], which is a commercially available textile with extremely high thermal and mechanical properties. In contrast to most aromatic compounds, which are formed in multistep reactions via the shikimate pathway [102], 3, 4-AHBA is biosynthesized via a unique pathway. In *Streptomyces griseus* cells, 3, 4-AHBA is formed from the glycolytic intermediate dihydroxyacetone phosphate and aspartate metabolite aspartate-semialdehyde in two-step aldol condensation reactions catalyzed by the gene products of *griI* and *griH*, respectively (Fig. 1) [103]. Thus, the 3, 4-AHBA synthetic pathway can be engineered in other microorganisms by introducing the corresponding heterologous genes, thereby potentially permitting high 3, 4-AHBA productivity from renewable feedstocks. As an example, *C. glutamicum* heterologously expressing the *griI* and *griH* genes produced 1.0 g/L 3, 4-AHBA from sweet sorghum juice [104].

Aromatic polyimides are alternative building blocks for high-performance bio-based polymers due to their excellent thermo-mechanical performance, high chemical stability, and low coefficient of thermal expansion. A phenylpropanoid derivative of 4-aminocinnamic acid was produced by the bioconversion of the non-standard amino acid 4-aminophenylalanine using a recombinant *E. coli* strain [92]. Bio-based polyimide was subsequently produced from a photodimer of 4-aminocinnamic acid through a chemocatalytic reaction. The resulting polyimide films exhibited ultra-high thermal resistance with a glass transition temperature of over 250 °C (the highest value for all bio-based plastics reported to date); these films also had high tensile strength and Young’s modulus [92]. The 4-aminocinnamic acid precursor 4-aminophenylalanine can be produced from glucose by microbial fermentation [105], suggesting that the fermentation and subsequent bioconversion of 4-aminophenylalanine can be produced using 4-aminocinnamic acid as a building-block material for the synthesis of bio-based polyimides from renewable sugars.

Compared to current aliphatic polymers, emerging bio-based aromatic polymers are value-added molecules with high thermal and mechanical properties; these polymers therefore might serve as engineering plastics. Further developments to increase the compatibility of aromatic compounds for bioprocessing will be needed to achieve high productivity of aromatic bio-monomers from renewable feedstocks.

## Conclusions

The present review aimed to provide a broad view of metabolic engineering strategies for producing building block chemicals for use in generating aliphatic polymers. We further described the current state of knowledge for the production of building block chemicals of next-generation, high-performance aromatic polymers. As described above, advances in metabolic engineering have markedly improved the productivities and yields of microbially produced polymer building blocks. Following the success of industrial l-lactic acid production through microbial fermentation, several bio-based approaches for succinic acid synthesis recently have been commercialized [55]. However, further improvements related to productivity and yield are required for many chemicals, particularly those that are synthesized via peripheral metabolic pathways. To realize this goal, novel methods for the rational design and optimization of enzymes and transporters to improve substrate specificity and reaction rates likely will be necessary. These developments are expected to enable efficient redirection and acceleration of carbon flux towards the target chemicals and extracellular secretion, respectively.

## Additional file

10.1186/s12934-016-0411-0 Engineering target genes covered in this review.

## Abbreviations

LDH: lactate dehydrogenase
NADPH: nicotinamide adenosine dinucleotide phosphate
ODH: 2-oxoglutarate dehydrogenase
PLLA: poly-L-lactic acid
TCA: tricarboxylic acid
