# Supplementary material for: Engineering cell factories for producing building block chemicals for bio-polymer synthesis
Source: Microb Cell Fact. 2016 Jan 21;15:19. doi: 10.1186/s12934-016-0411-0 (PMC4722748; doi:10.1186/s12934-016-0411-0)
Supplement: Supplementary file 1 — 10.1186/s12934-016-0411-0 Engineering target genes covered in this review. [file 12934_2016_411_MOESM1_ESM.docx]

Additional File 1: Table S1. Engineering target genes covered in this review

| Gene | | Enzyme | E.C. number | Target chemicals |
| --- | --- | --- | --- | --- |
| Glycolysis | |  |  |  |
|  | *ptsG* | Phosphotransferase system, glucose-specific  IIABC component | 2.7.1.69 | Succinic acid |
|  | *glk* | Glucokinase | 2.7.1.2 | Lactic acid |
|  | *pfk* | 6-Phosphofructokinase | 2.7.1.11 | Lactic acid |
|  | *fbp* | Fructose-1,6-bisphosphatase | 3.1.3.11 | Cadaverine |
|  | *fba* | Fructose-bisphosphate aldolase | 4.1.2.13. | Lactic acid |
|  | *gapA* | Glyceraldehyde 3-phosphate dehydrogenase | 1.2.1.12 | Lactic acid |
|  | *tpi* | Triosephosphate isomerase | 5.3.1.1 | Lactic acid |
|  | *ppsA* | Phosphoenolpyruvate synthase | 2.7.9.2 | Lactic acid |
|  | *ppc* | Phosphoenolpyruvate carboxylase | 4.1.1.31 | Lactic acid/ Succinic acid |
|  | *sfcA* | Malic enzyme | 1.1.1.38 | Succinic acid |
|  | *pepck*/*pck* | Phosphoenolpyruvate carboxykinase | 4.1.1.32/4.1.1.49 | Succinic acid/ Cadaverine |
|  | *pyc* | Pyruvate carboxylase | 6.4.1.1 | Succinic acid/ Cadaverine |
| Pentose phosphate pathway | | |  |  |
|  | *zwf* | Glucose-6-phosphate dehydrogenase | 1.1.1.49 | Cadaverine |
|  | *pgl* | 6-Phosphogluconolactonase | 3.1.1.31 | Cadaverine |
|  | *tkt* | Transketolase | 2.2.1.1 | Cadaverine |
|  | *tal* | Transaldolase | 2.2.1.2 | Cadaverine |
| TCA cycle | |  |  |  |
|  | *gltA* | Citrate synthase | 2.3.3.1 | Succinic acid |
|  | *icd* | Isocitrate dehydrogenase | 1.1.1.42 | Putrescine |
|  | *odhA* | 2-Oxoglutarate dehydrogenase E1 component | 1.2.4.2 | Putrescine |
|  | *frdA* | Fumarate reductase | 1.3.5.4 | Lactic acid |
|  | *sdhA* | Succinate dehydrogenase | 1.3.5.1 | Adipic acid |
| Amino acid biosynthesis | | |  |  |
|  | *aspC* | Aspartate aminotransferase | 2.6.1.1 | Succinic acid |
|  | *argB* | Acetylglutamate kinase | 2.7.2.8 | Putrescine |
|  | *argC* | N-acetylglutamylphosphate reductase | 1.2.1.38 | Putrescine |
|  | *argD* | Acetylornithine aminotransferase | 2.6.1.11 | Putrescine |
|  | *argE* | Acetylornithine deacetylase | 3.5.1.16 | Putrescine |
|  | *argF* | Ornithine carbamoyltransferase chain F-monomer | 2.1.3.3 | Putrescine |
|  | *argI* | Ornithine carbamoyltransferase chain I-monomer | 2.1.3.3 | Putrescine |
|  | *speC* | Ornithine decarboxylase | 4.1.1.17 | Putrescine |
|  | *lysC* | Aspartate kinase | 2.7.2.4 | Cadaverine |
|  | *hom* | Homoserine dehydrogenase | 1.1.1.3 | Cadaverine |
|  | *dapA* | Dihydrodipicolinate synthase | 4.3.3.7 | Cadaverine |
|  | *dapB* | Dihydrodipicolinate reductase | 1.17.1.8 | Cadaverine |
|  | *ddh* | Diaminopimelate dehydrogenase | 1.4.1.16 | Cadaverine |
|  | *lysA* | Diaminopimelate decarboxylase | 4.1.1.20 | Cadaverine |
| Other enzymes | |  |  |  |
|  | *budA* | α-Acetolactate decarboxylase | 4.1.1.5 | Succinic acid |
|  | *ldhA* | Lactate dehydrogenase | 1.1.1.27 | Succinic acid |
|  | *mgsA* | Methylglyoxal synthase | 4.2.3.3 | Succinic acid |
|  | *adhA* | Alcohol dehydrogenase | 1.1.1.1 | Succinic acid |
|  | *pflB*/*tdcE* | Pyruvate formate lyase | 2.3.1.54 | Succinic acid |
|  | *pta* | Phosphotransacetylase | 2.3.1.8 | Succinic acid |
|  | *cat* | Acetyl-CoA:CoA transferase | 2.8.3.18 | Succinic acid |
|  | *ack*/*tdcD* | Acetate kinase | 2.7.2.1 | Succinic acid |
|  | *pqo*/*poxB* | Pyruvate oxidoreductase | 1.2.5.1 | Succinic acid |
|  | *mgsA* | Methylglyoxal synthase | 4.2.3.3 | Succinic acid |
|  | *paaJ* | β-Ketoadipyl-CoA thiolase | 2.3.1.174 | Adipic acid |
|  | *hbd*/*paaH1* | 3-Hydroxybutyryl-CoA dehydrogenase/ Hydroxyacyl-CoA dehydrogenase | 1.1.1.157/1.1.1.35 | Adipic acid |
|  | *crt*/*ech* | Crotonase/ putative enoyl-CoA hydratase | 4.2.1.17 | Adipic acid |
|  | *ter* | Trans-enoyl-CoA reductase | 1.3.1.38 | Adipic acid |
|  | *ptb* | Phosphate butyryltransferase | 2.3.1.19 | Adipic acid |
|  | *buk1* | Butyryl kinase | 2.7.2.7 | Adipic acid |
|  | *speE* | Putrescine/cadaverine aminopropyltransferase | 2.5.1.16 | Putrescine/ Cadaverine |
|  | *puuA* | Glutamate-putrescine ligase | 6.3.1.11 | Putrescine/ Cadaverine |
|  | *speG* | Putrescine/cadaverine acetyltransferase | 2.3.1.57 | Putrescine/ Cadaverine |
|  | *snaA* | Putrescine acetyltransferase | 2.3.1.57 | Putrescine |
|  | *Ncgl1469* | Cadaverine acetyltransferase | 2.3.1.57 | Cadaverine |
|  | *ygjG* | Putrescine/cadaverine aminotransferase | 2.6.1.82 | Cadaverine |
|  | *cadA*/*ldcC* | Lysine decarboxylase | 4.1.1.18 | Cadaverine |
|  | *griH* | 3-Amino-4-hydroxybenzoic acid synthase | 4.1.99.20 | 3-Amino-4-hydroxybenzoic acid |
|  | *griI* | DhnA type aldolase | – | 3-Amino-4-hydroxybenzoic acid |
|  | *pprA* | Phenylpyruvate reductase | 1.1.1.237 | Phenyllactic acid |
|  | *pal* | Phenylalanine ammonia-lyase | 4.3.1.24 | Cinnamic acid |
| Transporters | |  |  |  |
|  | *sucE* | Succinate exporter | – | Succinic acid |
|  | *puuP* | Putrescine/ Cadaverine importer | – | Putrescine/ Cadaverine |
|  | *potE* | Putrescine/ornithine antiporter | – | Putrescine |
|  | *cadB* | Cadaverine exporter | – | Cadaverine |
|  | *cgmA* (*cgl2893*) | Putrescine/ Cadaverine exporter | – | Putrescine/ Cadaverine |
